# Supplementary material for: Functional comparison of human and murine equilibrative nucleobase transporter 1
Source: PLoS One. 2024 Oct 3;19(10):e0311519. doi: 10.1371/journal.pone.0311519 (PMC11449324; doi:10.1371/journal.pone.0311519)

Original Blots

PONE-D-24-27708 Manuscript

Original Blots from PCR results reported in Figure 3

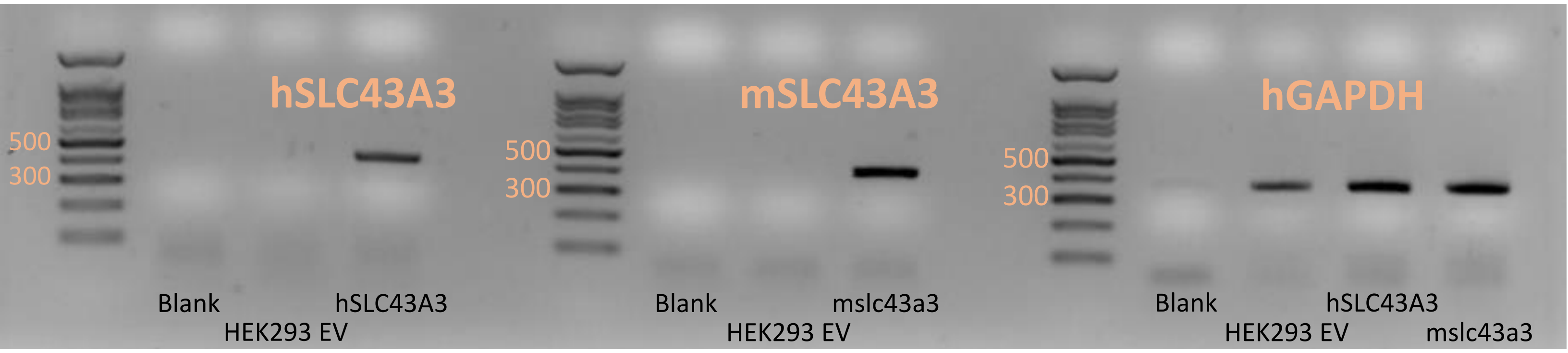

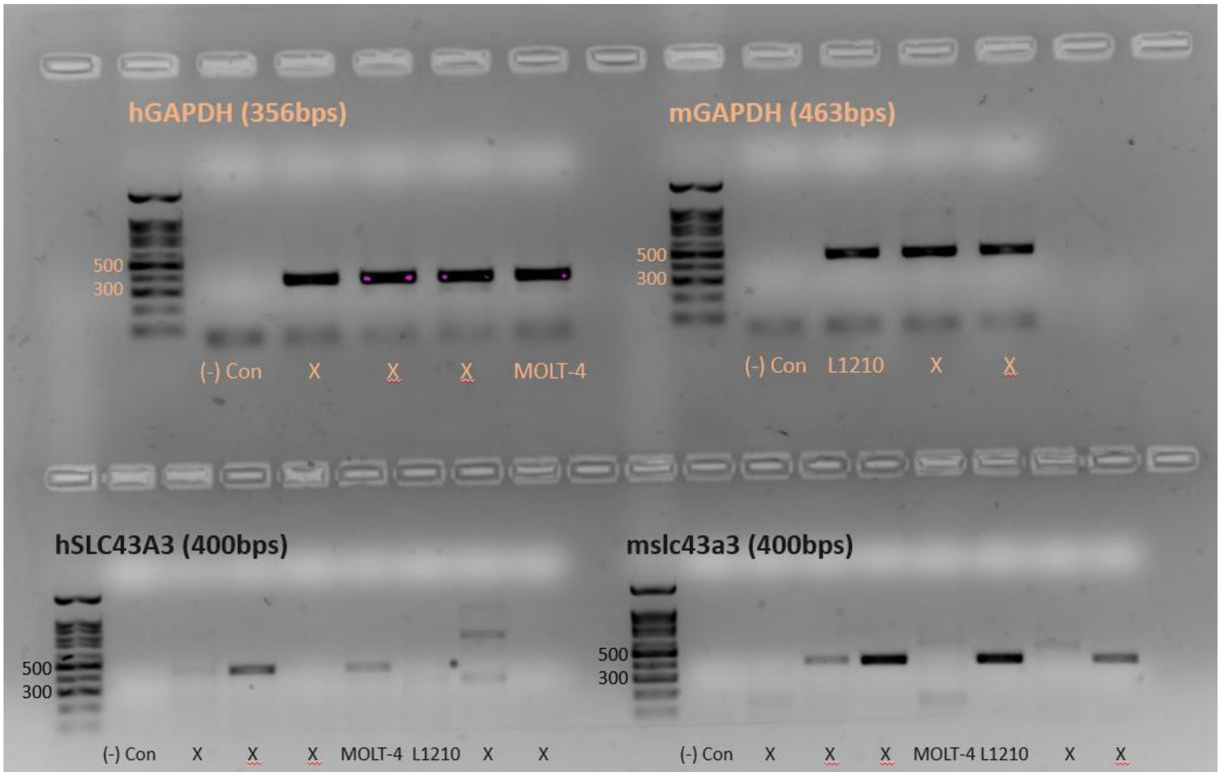

X – indicates not used in manuscript

Original western blots for results reported in Figure 4A

X – indicates not used in manuscript

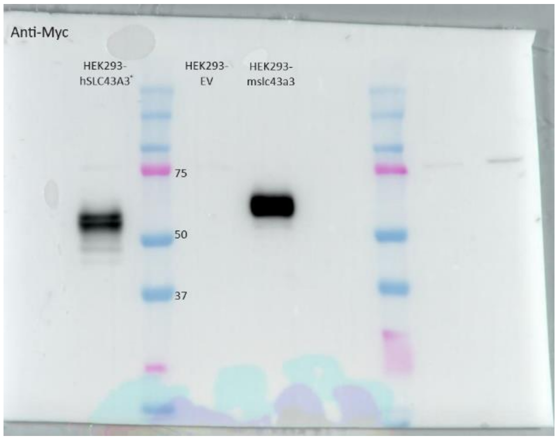

Anti-Myc

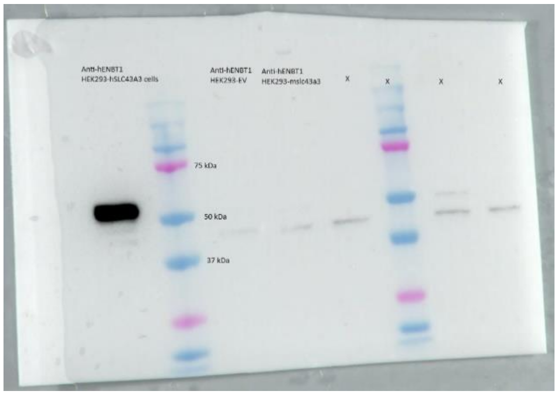

Anti-hENBT1

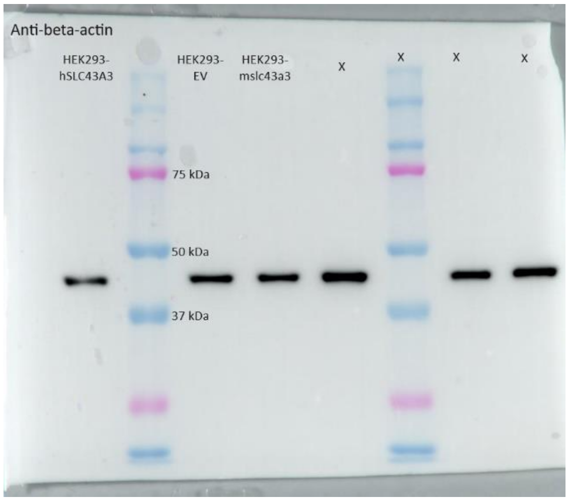

Anti-Beta Actin

Original western blots for results shown in Figure 4C

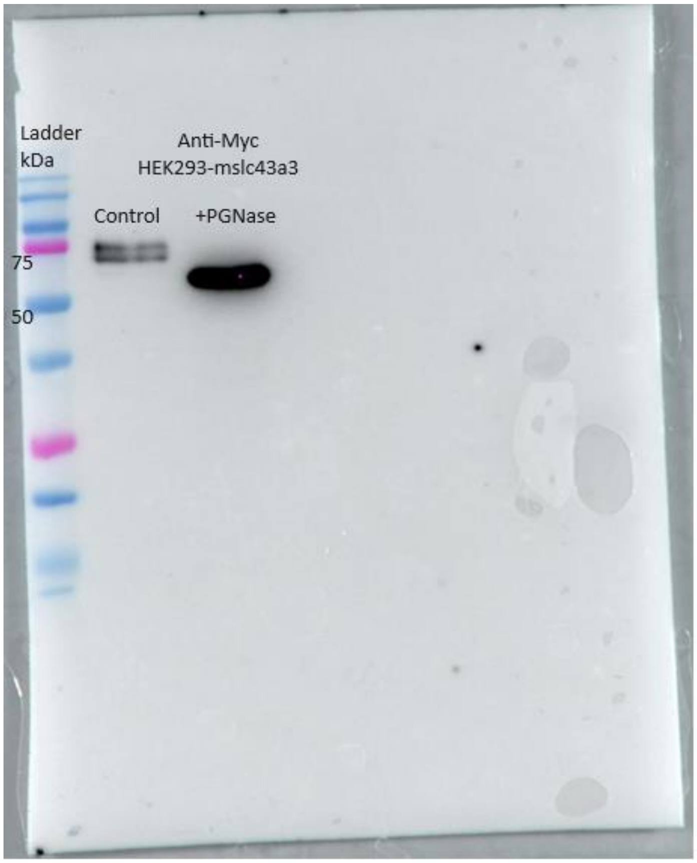

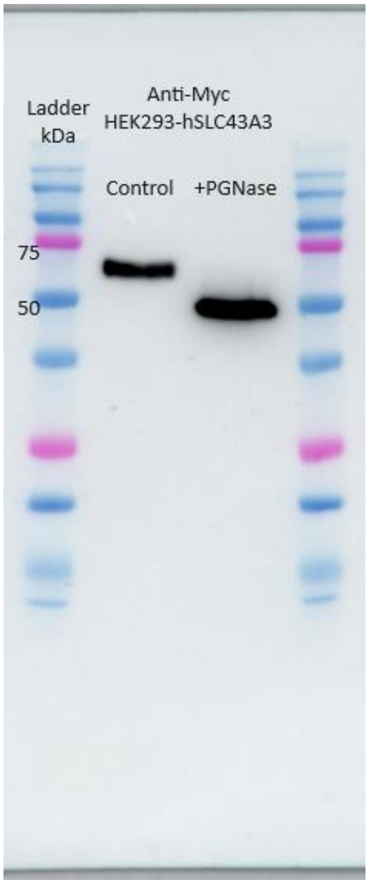

Supplement: S1 Raw images — (PDF) [file pone.0311519.s001.pdf]
