# Supplementary material for: Functional comparison of human and murine equilibrative nucleobase transporter 1
Source: PLoS One. 2024 Oct 3;19(10):e0311519. doi: 10.1371/journal.pone.0311519 (PMC11449324; doi:10.1371/journal.pone.0311519)
Supplement: Attachment — Submitted filename: Response to reviewers.pdf [file pone.0311519.s002.pdf]

RESPONSE TO REVIEWERS

We have provided the reviewers' comments in italics and our response follows each comment in bold type.

Reviewer 1: *General comments: Seems some key references are missing from this submission, please review submission for opportunities to include additional relevant citations and ensure correct format. One example is the 2004 paper from Baldwin, Cass, and others on SLC29s that helps support linkage of transporter expression levels on drug efficacy and toxicity. (Note: This reviewer is not affiliated with the above paper).*

The 2004 Baldwin et al paper on the SLC29 transporter family is already referenced in the manuscript with respect to the low affinity transport of 6-MP by ENT1 and ENT2. We have now also used it as a reference to support the discussion on the effect of DY on the impact of 6-MP on cell viability, as suggested (end of discussion section).

Narrative flow can be improved by breaking down longer sentences and paragraphs. Ensure consistent use of abbreviations and define them upon first use. The Discussion section is well written overall yet raises several key questions about 6-MP and adenine interaction with, and transport by, ENBT1 from both human and murine systems. It seems addressing these questions is an objective of follow-on publications leveraging the model presented. For model validation, the current work is sufficient to support more complete studies.

We have made some style edits throughout to enhance clarity and flow.

Line 7 -8: missing punctuation after myelotoxicity

Fixed

Line 17 – 19: vague on details, what is meant by "in silico" in this instance? Is this just a pairwise sequence alignment or more?

Just pairwise sequence analysis. That section has been condensed and made clearer.

Also, the final abstract statement seems a bit broad in terms of "models" and "actions, adverse effects, and pharmacokinetics". Is the intent to specifically study ENBT1-mediated action or broader impact beyond specific interactions of the transporter with substrates? The current manuscript doesn't support mouse models as a general tool for studying disposition and impact of 6-MP relative to human pathology.

We agree that this statement was too broad in scope. It has been changed to: "*Therefore, data obtained from mouse models where ENBT1 is disrupted or modified may provide clinically relevant insights on its role in modulating the actions of 6-MP*"

Line 24: "various cancers including acute lymphoblastic leukemia (ALL) and autoimmune conditions such

like inflammatory bowels disease (IBD) and ulcerative colitis [1]." should be "various cancers including acute lymphoblastic leukemia (ALL) and autoimmune conditions such as inflammatory bowel disease (IBD) and ulcerative colitis [1]."

Fixed

Line 27: "minimization off-target toxicities" should be "minimization of off-target toxicities."

Fixed

Line 40: "our lab further characterized ENBT1 in stably transfected human embryonic kidney cells 293 (HEK293) cells" should be "our lab further characterized ENBT1 in stably transfected human embryonic kidney 293 (HEK293) cells."

Fixed

Line 58: extra space needed "6 -7" vs. "6 – 7"

Fixed

M&M: Overall, the Materials and Methods are well written with specific detail to reproduce experiments. Well done.

Thank you.

Line 99: Suggest adding an approved IACUC protocol number for the associated animal studies with this statement. Also, "using protocols approved by the Animal Care Committee of the Faculty of Medicine & Dentistry University of Alberta" should be "using protocols approved by the Animal Care Committee of the Faculty of Medicine & Dentistry, University of Alberta."

Added the animal use protocol number. We are not sure what else the reviewer wanted us to change – there was already a comma after "… Dentistry, …"

Line 110: More specific detail on cell culture protocol would be beneficial including concentrations and durations where needed.

We have provided more detail on the cell culture procedure

Line 225 – 228: The statement "The highest expression levels of slc43a3 were seen in the lung followed by heart liver kidney and spleen with relatively lower expression in duodenum and jejunum." would benefit from quantitative data or a reference figure/table. This statement refers to Figure 3 yet this is just a bar

histogram with SEM. Are differences in expression significant between tissue types? Can specific values +/- SEM be added here in parentheses in support of the figure?

We have done the statistical analysis on this data set, indicated statistical differences on the graph, and added relative difference information to the text of the results.

Line 410 – 412: "Overall SLC43A3/hENBT1 and slc43a3/mENBT1 had similar effects on 6-MP and 6-TG induced decreases in cell viability further suggesting the human and murine variants of ENBT1 are functionally similar." – Can this be expanded to denote potential clinical significance or broader impacts? Last line of the abstract focuses on "actions, adverse effects, and pharmacokinetics" – there is value in connecting these two at this point.

We have moved this summary statement to the "Conclusion" section, and expanded that final section to cover the potential of the mouse model for conducting experiments to provide insights on the variability seen clinically in 6-MP pharmacokinetics and incidence of adverse effects.

"Conclusion": As written, this conclusion seems like a missed opportunity to expand. Either the broader impacts language in the abstract and parts of manuscript should be dialed back or this section can be developed a bit more. This reviewer supports the latter.

Addressed above

Figures: Figures need additional work and polishing.

Figure 1 is cumbersome to understand meaning or intent without work. Other publications in this field have used structures to convey similar changes and pathways where one can more easily see conversions. As shown, this version is difficult to follow and many of these conversions are not relevant to the manuscript as written or discussed.

We have removed Figure 1 in the revised manuscript (as recommended by Reviewer 2)

Figure 2 is full page yet still impossible to define detail noted in the legend. Amino acids are identified within circles but unreadable. Figure is blurry even on screen within the PDF. It's impossible to see or understand the red markings as noted in the figure legend. It's important to include this figure – that is clear. But, the current form adds minimal value. Consider changing this graphic to more clearly show differences with larger typeface. Another option is, since the topology is almost identical, just show one panel with topology and a sequence alignment below noting differences in sequences.

We have revised figure 2 to show only the mouse ENBT1 topology, and added the mouse/human sequence alignment below displaying differences in sequences. Thank you for this suggestion.

Figure 3 would benefit from noting significance between tissues – particularly lung relative to heart – spleen.

This has been done and the figure revised appropriately

Figure 6 – What specifically does "from 6 independent experiments conducted in technical duplicate" mean for transport assays? The mechanics of doing replicates isn't defined in the transport assay or statistical analysis sections.

The technical replicates were done simultaneously within one experiment and averaged, and this was repeated several times with different cell isolations to achieve the final sample size (n). I have added this detail to the statistical analysis section and simplified this in the figure legends.

General comment on fits – it would be beneficial to include fit values (e.g., Ki or Vmax) within graphs for easy visual comparison. As written, this information is within the main text (results) but not figure legends. One suggestion is to also add these values directly within figures as well.

The Km and Vmax values have been added to the figures.

Reviewer #2: The objective of the studies in this manuscript was to establish that the mouse equilibrative nucleobase transporter 1 or mENTB1 (Slc43a3) is similar in its tissue distribution and function towards 6-mercaptopurine (6-MP) as the human counterpart hENTB1 (SLC43A3). These studies are intended to validate the utility of a mouse null mutant model (Slc43a3-) for mENTB1 that will be used to follow 6-MP pharmcokinetics. Both hENTB1 and mENTB1 share a similar topology and share 73.4% identity at the primary sequence level. Using quantitative RT-PCR analyses on several different tissue types isolated from C57BL/6 mice the authors demonstrate that the tissue distribution for Slc43a3 expression is similar to that observed for SLC43A3 in human tissue. Kinetic analyses on HEK293 cells expressing the coding sequences SLC43A3 (hENTB1) and Slc43a3 (mENTB1) indicated that the kinetic properties of these transporters are highly similar with respect to the ligands, adenine and 6-mercaptopurine. Overall, the studies in the manuscript are well executed and thorough. However, there are a few minor points that should be addressed to improve the overall clarity of the manuscript.

1. Consider removing Figure 1. I'm not sure that it is relevant or adds much to the study. If the figure is still included, it might be useful to indicate which activities are primarily affected by genetic variations.

We agree that it did not really add much, and have removed Figure 1 from the revised manuscript.

2. Is anything known about genetic variations in SLC43A3 and 6-MP toxicity? The authors allude to a role for this transporter in 6-MP bioavailability in the introduction, but I'm confused whether genetic variations in this transporter have been described. Please clarify this point in the introduction.

There are several known polymorphisms of the SLC43A3 gene. However, none of them have been assessed with respect to how they affect transport activity or distribution. This is an area that is ripe for future investigation. I have added this information in the conclusion section of the manuscript.

3. The presence of the polyglutamine tract in mENTB1 (slc43a3) is interesting and potentially could provide a site of interaction between mENTB1 transporters, or with other cytosolic proteins. What is known about the quaternary structure for mENTB1 and is it different than that of hENTB1? Perhaps the authors could provide some additional discussion about the significance of the polyglutamine tract in the manuscript.

There is no information available concerning the quaternary (or even tertiary) structure of ENBT1. We agree that the presence of the polyglutamine tract may lead to additional protein interactions in the mouse ENBT1 which could affect its regulation and trafficking This has been added to the revised discussion. However, since it is not present in human ENBT1, interactions involving such a polyglutamine tract are not likely to be clinically relevant.

4. Figure 3 and Ln 227: I suggest changing the order in the text of the tissues to match the order in the figure (heart, kidney, liver, and spleen). Were the differences in the relative expression levels statistically significant? Please include that information in the figure or text. Additionally, please specify that the

tissue distribution is for the mouse strain C57BL/6 in the text. Is this the same mouse strain used to create the null mutant? If so, please include in the discussion.

We have changed the tissue order in the text, and updated Figure 3 (now Figure 2) to show statistical differences. The tissue distribution data is indeed from the C57BL/6 strain, as stated in the first sentence of the gene expression methods section, and this is the strain in which the null mutant is being made.

5. Ln 237 - 239: In text mention that qualitative PCR and immunoblot were performed using cDNA and lysates from HEK293 transfected cells, as well as the MOLT-4 and L1210 cell lines and this data is shown in figure 4. However, the data in Figure 4 shows just the PCR results for the HEK293 transfected cell lines.

We repeated this PCR assay/gel to include all of the *SLC43A3* data (using the same primers) on a single gel, and the *slc43a3* expression data in L1210 cells on a separate gel (due to different primers being used). The MOLT-4 and L1210 qPCR data is now shown in Figure 3. Since we do not have a good mouse ENBT1 antibody, we cannot do an immunoblot for the murine L1210 cells, and due to this lack we felt a MOLT-4 immunoblot comparison would be superfluous. The text has been changed to better describe what was done.

Additionally, please indicate the primer pairs used for the PCR analysis, as well as the PCR conditions, in the figure legend. If these are the same as for the qPCR, please indicate as such.

This information is all presented in the methods section with the primers shown in Table 1. The primers were different for the qPCR (using the Roche Lightcycler) and the standard 'qualitative' PCR, as indicated in Table 1 (now referenced in the legend). Does the reviewer mean to additionally add them to the figure legend? This seems to be redundant to us.

6. Ln 241 -243: The data or citation for the expression of SLC43A3 in MOLT-4 cells and Slc43a3 in L1210 cells doesn't appear to have been included?

Those data have now been included in the revised Figure 3.

7. Lns 273-275: Consider moving the Km and Vmax values (as well as the Ki values) to a table format for conciseness and ease of comparison.

Rather than using a table to summarize the values, we have added them to the figures themselves (this was suggested by Reviewer 1). The Ki values are now shown on the inhibition profiles in Figure 6. The Km and Vmax values are shown on Figures 5 and 8.

8. Figure 7B and Lns 290-295: I'm intrigued by the inhibition data using 6-MP as an inhibitor of adenine uptake. The data suggest that the inhibition mechanism might be more complex than just competitive inhibition. I would suggest that the authors provide a more detailed discussion for the underlying reasons for this observation and expand on the comment in the discussion that it may be due to differing substrate-ENTB1 Koff values.

Without knowing more about the translocation process used by ENBT1, any further discussion would just be speculation at this point. That is why we kept that part of the discussion fairly limited. We have expanded on this a bit though using the assumption that it is some sort of alternating-access mechanism that is being used (as is common for other SLC proteins) with the substrate binding site alternating between being accessible to the intracellular or extracellular environments.

9. Figure 8: The data points for 6-MP uptake in MOLT-4 and L1210 cells show substantial deviation and are not particularly convincing of a linear correlation between 6-MP uptake with time. Indeed, in the MOLT-4 cells the difference in 6-MP uptake doesn't seem to change between 2 sec and 20 sec? Does this reflect solely binding of 6-MP to the transporter and not uptake per se? I'm curious if the authors have tried extending the time course (to say 5 or 10 mins) and what those results reflected?

It is challenging technically to get those short time points in this type of assay – hence the variability. However, it is substrate transport, not binding. This can be seen from the fact that the intracellular concentration of the radiolabeled substrate at steady-state is similar to the extracellular concentration added (~30 µM; see Figure 7), as would be expected for a facilitated diffusion transporter such as ENBT1. The rate of cellular accumulation of 6-MP can also be slowed down with the inclusion of a different substrate such as 6-thiopurine. In addition, the substrate efflux kinetics are similar to the influx kinetics (see Ruel et al., 2019).

Using longer time points is problematic as these substrates (both adenine and 6-MP) are phosphorylated metabolically, thereby trapping the radiolabel in the cell as impermeable metabolites that would maintain an inward substrate gradient. This would result in the kinetics reflecting that of the rate limiting enzyme as opposed to the transporter itself. We have shown in previous publications that this transporter is extremely efficient, and that even a 2 second incubation time doesn't truly reflect 'initial rates' of transport. This is a limitation of the transport assay methodology used.

10. Figures 8A and 9A: Consider changing the symbols in these figures to open and closed symbols to more clearly differentiate between the total and non-mediated ENTB1 uptake. When the symbols overlay it is difficult to differentiate between symbols of the same color and shape.

We have modified those figures as suggested.

11. Figures 10 and 11: The authors speculate that the difference between the biphasic and monophasic EC50 curves for TG and 6-MP reflects their different routes of metabolism. I'm a little confused by that interpretation. Wouldn't an EC50 values in the same population be monophasic regardless of which intracellular targets the drug interacts with? I would suggest instead that this reflects different subpopulations within the experiment, which seems quite likely for Figure 10 when dealing with transfected cells.

Both 6-MP and TG were tested on the same cell preparations, often in parallel. So it does not reflect different subpopulations of cells in the two cases. We think what is happening is that 6-TG enters the metabolic pathway well downstream from 6-MP, and is directly phosphorylated into 6-TG nucleotides. This may make it more cell cycle independent. 6-MP on the other hand has a number of intermediate

metabolites (e.g. 6-methylmercaptopurine) that are known to also impact RNA synthesis. It may be that 6-MP is leading to a decrease in cell viability in actively dividing cells, but is less active in quiescent cells at the time of addition to the cells. We have tried to investigate this using cell cycle analysis prior to and after 6-MP treatment, but the results have been ambiguous. We have expanded on this a bit in the discussion though.
